# Surgery

**Published:** 1907-06

**Authors:** C. A. Morton


### SURGERY.

Several papers have been published during the last few years[2] on **delayed anæsthetic poisoning,** and such a condition is now well recognised, and comes, of course, under the observation of the surgeon in charge of the case, and not the anæsthetist. As the symptoms are not pathognomonic, it may be extremely difficult to say in some cases whether they have been caused by the anæsthetic or not, and it is highly probable that in several cases in which they have been attributed to it they have been due to other causes. In many of the cases of late anæsthetic poisoning, acetone or diacetic acid, or both, have been found in the urine, and there is often a smell of acetone in the breath; but acetone has been frequently found in the urine after the administration of an anæsthetic without symptoms,[3] and it is stated by Guthrie to be present in "starvation, malignant cachexia, peritonitis, gastric ulcer and other abdominal disorders, sepsis, pneumonia, after poisoning by phloridzin and morphine," as well as in diabetes, and he says "it may be artificially induced by the injection of fat." Indeed, it seems to me probable that if carefully looked for it would be found in the urine of healthy persons fairly often.

[2] Guthrie, *Lancet*, 1894, i. 193; 1903, ii. 10; 1905, ii. 583.

[3] Hubbard, *Boston M. and S. J.*, 1905, clii. 744; abstract in *Lancet*, 1905, ii. 234.

Kelly[1] found acetone and diacetic acid in the urine of patients on admission to hospital suffering from various diseases who had not had an anæsthetic, and without any symptoms like those of late chloroform poisoning. Moreover, symptoms such as occur in these cases of late chloroform poisoning may occur without any anæsthetic or operation, and may be fatal,[2] and the condition present in paroxysmal vomiting of children is a closely allied one. Again, sepsis may cause similar symptoms, and it would be almost impossible to say in a septic case that they were due to delayed anæsthetic poisoning rather than to the absorption of a septic toxine, though not necessarily one producing pyrexia. It is only in cases in which the patient is in good general health at the time of the operation, and this an aseptic one and unattended by shock or absorption of carbolic acid from the skin, that we can regard the anæsthetic as the cause of the symptoms. Chloroform has been mainly responsible for such symptoms, but there are a few cases in which they have followed the administration of ether.[3] In carboluria the peculiar conditions of the urine would, of course, indicate the nature of the case, but the symptoms closely resemble those of late anæsthetic poisoning. Some seem to think those of fat embolism do also, but they certainly do not in a typical case of fat embolism.

The symptoms of late anæsthetic poisoning are described by Guthrie[4] as profuse and repeated vomiting, the vomit eventually resembling the dregs of beef-tea, restlessness, excitement, delirium, alternating into periods of apathy, occasionally jaundice, and unconsciousness deepening into coma. The symptoms come on about twelve hours after the anæsthesia, and in the interval the patient may seem to be progressing favourably. Death may occur in twelve hours, but not usually until the fifth day. There is not usually any rise of temperature, but it may be very high. Albumin may be present, and casts may be found in the urine also. I have already referred to the smell of acetone in the breath.

In many of the fatal cases extreme fatty change has been found in the liver, and also a similar change in the heart, in the muscles, and in the kidneys, and no other condition has been discovered associated with this.

It is thought by most of those who have studied this subject that the fatty change is produced by the chloroform, and in some way, and at any rate to some extent, is the cause of death; but Guthrie holds that it precedes the anæsthesia, and is only augmented by it, but that this augmentation is in some way the cause of the symptoms. If such fatty change, found after the administration of chloroform, were caused by it alone, both the fatty

[1] Kelly, *Ann. Surg.*, 1905, xli. 161.

[2] Brackett, Stone and Low, *Boston M. and S. J.*, 1904, cli.; abstract in *Lancet*, 1904, ii. 846.

[3] Favill, *J. Am. M. Ass.*, 1905, xlv. 691. [4] Guthrie, *Lancet*, 1905, ii. 583.

change and the resulting symptoms ought to occur in proportion to the duration of the administration ; but they do not. Indeed, if such fatty change were the sole cause and were due to the anæsthetic, cases of late anæsthetic poisoning should only occur after very prolonged anæsthesia (cases, in fact, in which an excessive dose has been given), as it is such a very rare condition. If, therefore, the fatty change is due to the chloroform, and in any degree causes the symptoms, there must be also some other factor at work, and we are quite ignorant as to what this is, for there seems to be no evidence to support Guthrie's view that fatty degeneration of the liver precedes the administration of the chloroform, though if it did the effect of the chloroform would be readily explained, for it would act " as the last straw," as it has been expressed. There is no doubt an extremely fatty liver may be present in children who have had no anæsthetic. Dr. John Thomson, of Edinburgh, has seen very extreme examples in children dying of broncho-pneumonia.[1]

We know from experiments on animals[2] that subcutaneous injection and inhalation of chloroform will produce fatty degeneration of the liver and other organs. In some of the experiments a control examination of the liver was made under ether at an earlier period. Ether injected subcutaneously did not produce this fatty change in the liver, yet some of the fatal late anæsthetic poisoning cases have been after ether, and fatty changes were found in the liver, kidneys and muscles.[3] Guthrie regards such cases as proof of pre-existing fatty change in the liver[4]. But then, if ether does not markedly increase this, how could such fatty change, accelerated by the ether, have been the cause of death ? It must need a very light straw indeed, as the last one, if the ether is to be blamed at all.

In chronic tuberculosis fatty changes in the liver are quite common, yet late anæsthetic poisoning is not specially common in such cases.

It cannot then be regarded as proved that the fatty change found in the liver, heart and kidneys is by itself really the cause of death in these cases. Even granting that it is in some way connected with the death, in what way it is so is still quite uncertain. Neither is the relation of the symptoms to the presence of acetone and diacetic acid clear. In fact, at the present time the whole subject is very obscure.

The treatment adopted by some in diabetic coma has been recommended for this condition—saline transfusion, and the administration of bicarbonate of soda. It would seem that if it

[1] Stiles and McDonald, *Scottish M. and S. J.*, 1904, xv. 97.

[2] Stiles and McDonald, *loc. cit.;* Ungar and Junkers, *Über fettige Entartung in Folge von Chloroform-inhalationen*, Bonn, 1883 ; Ostertag, *Arch. f. path. Anat.*, 1889, cxviii. 250 ; Schenk, *Ztschr. f. Heilk.*, 1898, xix. 393.

[3] Carmichael and Beattie, *Lancet*, 1905, ii. 437.

is an acid poisoning it is neither acetone nor diacetic acid, but a precursor of these—$\beta$ oxybutyric acid, which is the poisonous agent.

The publication by Mr. Barker of a paper on "**Spinal Anæsthesia**"[1] has brought the subject prominently before the minds of surgeons in this country, and it may be well to consider the value of this method of producing anæsthesia, as contrasted with the ordinary one. Mr. Barker does not suggest its general adoption in place of the usual method. He has tried it in 128 cases with a view to determine its value, and seems well pleased with it. It has been used in several thousand cases on the Continent by various surgeons.

There are three questions to be answered before we can form an opinion as to its value :—

1. Has it advantages over general anæsthesia as at present employed ? In answering this we must consider its relative risk to life, and the probability of unpleasant sequelæ.
2. What are the limitations as to its use ?
3. Is it certain in its action ?

There is now nothing unpleasant to the patient in general anæsthesia. When ether was given alone there undoubtedly was ; but ether preceded by nitrous oxide or ethyl chloride is not unpleasant to take, nor is chloroform. The after taste of ether is, however, often much complained of by patients. But, as Mr. Barker says, some patients have a great dread of a general anæsthetic. In what does this dread really consist ? If it is fear of death from the anæsthetic, then we can hardly represent to them that spinal anæsthesia is safer on the evidence at present before us. We should rather point out to them how seldom anyone dies from a general anæsthetic. If it is some unreasonable fear of losing consciousness, or dread of suffocation, then spinal anæsthesia seems most suitable in such cases. But many patients would, I think, prefer to lose consciousness, and to know nothing of what was going on. Mr. Barker tells us that those patients of his who had had both chloroform, and spinal anæsthesia, preferred the latter.

After a general anæsthetic vomiting is, of course, sometimes trying, though often absent, and headache may occur as well. With stovaine both headache and vomiting may occur, and the vomiting even occur during the operation (though this is very rare). In Mr. Barker's second series of fifty cases, some nausea or vomiting and headache were present in twenty, but only quite exceptionally was vomiting marked. These symptoms were usually removed by phenacetin and caffein, or a purgative. The after effects of stovaine in one hundred of Chaput's cases[2] were vomiting in ten

[1] *Brit. M. J.*, 1907, i. 665.

[2] "L'Anesthésie rachidienne à la Stovaine," *Arch. de Thérap.*, Nov. 15th, 1904 ; Chiene, *Scottish M. and S. J.*, 1906, xviii. 220.

(not always on the first day), nausea in thirteen, headache forty times (not always on day of operation), always relieved by application of ice. Neuralgia is said to have occurred in more than eighty cases, but it is not stated where. This also in thirty-five cases was later in its onset than the day of operation. When cocaine was used, these symptoms and pain in the back were often severe, and the after effects seemed worse than with general anæsthesia. With stovaine it is difficult to decide. They do not, at any rate, seem worse than the after effects of general anæsthesia.

The risk to life of spinal anæsthesia does not seem great, but there have been serious symptoms from its use, and I do not know that we are yet in a position to compare its risks with general anæsthesia. Tuffier up to 1904[1] had no bad results in 2,000 cases. At the German Surgical Congress, 1905,[2] Hermes, of Berlin, in reporting ninety cases of Sonnenberg's, said that a cold sweat with pallor and small pulse occurred in several abdominal operations under stovaine, and Preindlsberger, of Sarajewo, in 305 cases had six similar ones. Chaput[3] describes a case in which, after injection of stovaine from a "defective old stock," the pulse stopped, but returned with the administration of stimulants, and the use of artificial respiration. The case is quoted by Chiene[4]. Silbermark had used stovaine in 300 cases, and thought that with this drug, eucaine and tropacocaine, there was no danger; nor had Neugebauer observed any alarming symptoms in 480 cases. Bier stated that the use of an adrenal preparation with the stovaine limited its dangers considerably, and should always be used. Septic infection of the spinal canal seems never to have occurred in the experiences of these surgeons, and should not, of course, occur, but might if the method were employed by those unaccustomed to thorough methods of sterilisation. There is, at any rate, one case on record of persistent paraplegia following spinal cocainisation. At the Surgical Society of Paris a patient was shown who three days after the operation began to develop spinal symptoms, and they continued eight months later, when the case was reported.[5]

Almost all surgeons who have endeavoured to produce spinal anæsthesia have had failures, and some many failures, but it seems generally considered that this is due to faulty technique.

Mr. Barker says it may be better not to use this method for the very old, at all events for the present. But it is difficult to know at what age persons are to be regarded as very old. Bier[6] recommends it for the weak and aged; Hermes[7] for patients over 75.

Spinal anæsthesia has one distinct advantage, and that is that

[1] "Anesthésie rachidienne à la Stovaine," *Wien. klin. therap. Wchnschr.*, 1905, No. 15.

[2] *Ann. Surg.*, 1905, xlii. 942. [3] *Société de Biologie*, May, 1904.

[4] *Scottish M. and S. J.*, 1906, xviii. 220.

[5] *Lancet*, 1905, i. 677. [6] *Loc. cit.* [7] *Loc. cit.*

in an operation on the lower limb, such as a hip joint amputation, likely to be attended by shock, it tends to prevent it, just as Crile showed that the injection of cocaine into the large nerve trunks of the limbs would. In a patient much exhausted by hectic fever, I amputated just below the trochanters under stovaine spinal analgesia, and there was no shock at all; indeed, an hour after the operation his pulse was only 100°, whereas in the ward before he went up to theatre it had been 120° for more than twenty-four hours. The anæsthesia was perfect, and there was not the slightest nausea or headache after it. In cases in which the lungs or kidneys are not in a fit condition for general anæsthesia, spinal anæsthesia has also a distinct advantage.

Spinal anæsthesia above the umbilicus seems uncertain, but some surgeons have done operations on the upper abdomen with it, though others regard the anæsthetisation of this area as too uncertain. This is certainly a considerable disadvantage in abdominal surgery, for owing to uncertainty in diagnosis we may find we have to extend an incision begun for disease supposed to be in the lower abdomen into the upper, or the disease, though largely in the lower abdomen, may extend into the upper. For instance, what is diagnosed as acute appendicitis may turn out to be a perforating duodenal ulcer. Another disadvantage of spinal anæsthesia for some abdominal operations would be the inability to employ the Trendelenburg position, at any rate early in the operation. Speaking of their experience of spinal anæsthesia at the German Surgical Congress in 1905, both Bier and Silbermark did not recommend it at all for abdominal operations; but other surgeons have had a satisfactory experience of it in the lower abdomen.

There does not seem, then, any reason why spinal anæsthesia should replace general anæsthesia, except in cases in which the patient would prefer it, or in which there is so much collapse that we dread to give a general anæsthesia, and local anæsthesia would not be satisfactory, and in cases in which we specially fear severe shock, even though the patient is not collapsed; also when the heart, lungs, or kidneys are not in a condition for general anæsthesia. We must remember that if there is great abdominal distension it would not be easy to get the spine well curved forwards to make the lumbar puncture, and we must also realise fully that our time for performing the operation under spinal anæsthesia averages about an hour, though it may be increased by the addition of adrenalin to the fluid injected.

The technique of this method is fully described in Mr. Barker's paper.

C. A. MORTON.

